# Antinflammatory, antioxidant, and behavioral effects induced by administration of growth hormone-releasing hormone analogs in mice

**DOI:** 10.1038/s41598-019-57292-z

**Published:** 2020-01-20

**Authors:** Lucia Recinella, Annalisa Chiavaroli, Giustino Orlando, Claudio Ferrante, Guya Diletta Marconi, Iacopo Gesmundo, Riccarda Granata, Renzhi Cai, Wei Sha, Andrew V. Schally, Luigi Brunetti, Sheila Leone

**Affiliations:** 1 Department of Pharmacy, G. d’Annunzio University, Chieti, Italy; 2 Division of Endocrinology, Diabetes and Metabolism, Department of Medical Sciences, University of Turin and Città Della Salute e Della Scienza Hospital, Turin, 10126 Italy; 3 Veterans Affairs Medical Center, Miami, FL 33125 USA; 4 Division of Endocrinology, Diabetes and Metabolism, Department of Medicine, Miller School of Medicine, University of Miami, Miami, FL 33136 USA; 5 Division of Medical/Oncology, Department of Pathology, Sylvester Comprehensive Cancer Center, Miami, FL 33136 USA

**Keywords:** Prefrontal cortex, Neural circuits

## Abstract

Growth hormone-releasing hormone (GHRH) antagonist MIA-690 and GHRH agonist MR-409, previously synthesized and developed by us have demonstrated potent antitumor effects. However, little is known about the effects of these analogs on brain functions. We investigated the potential antinflammatory and antioxidant effects of GHRH antagonist MIA-690 and GHRH agonist MR-409, on isolated mouse prefrontal cortex specimens treated with lipopolysaccharide (LPS). Additionally, we studied their effects on emotional behavior after chronic *in vivo* treatment. *Ex vivo*, MIA-690 and MR-409 inhibited LPS-induced inflammatory and pro-oxidative markers. *In vivo*, both MIA-690 and MR-409 induced anxiolytic and antidepressant-like effects, increased norepinephrine and serotonin levels and decreased nuclear factor-kB, tumor necrosis factor-α and interleukin-6 gene expression in prefrontal cortex. Increased nuclear factor erythroid 2–related factor 2 expression was also found in mice treated with MIA-690 and MR-409. MIA-690 showed higher efficacy in inhibiting all tested inflammatory and oxidative markers. In addition, MR-409 induced a down regulation of the gene and protein expression of pituitary-type GHRH-receptor in prefrontal cortex of mice after 4 weeks of treatment at 5 µg/day. In conclusion, our results demonstrate anxiolytic and antidepressant-like effects of GHRH analogs that could involve modulatory effects on monoaminergic signaling, inflammatory and oxidative status.

## Introduction

Growth hormone (GH)-releasing hormone (GHRH) is a neurosecretory peptide produced by hypothalamic neurons which stimulates synthesis and release of GH in the anterior pituitary gland^1,2^. In addition to its recognized metabolic and endocrine effects, GHRH exerts also various effects on central and peripheral tissues such as brain, gastrointestinal tract, heart, kidney and retina^3–5^. In the pituitary, as well as in peripheral tissues, GHRH binds to pituitary-type GHRH-receptor (P GHRH-R), a G protein-coupled receptor which stimulates the adenylyl cyclase, cAMP and protein kinase A (PKA) cascade^6^, and to its splice variant (SV1)^1,7–9^.

Various GHRH receptor agonist and antagonist peptides have been synthesized by us and other groups and studied for their biological activity^1,10–16^. In particular, the novel GHRH antagonists of the Miami (MIA) series, MIA-690 and MIA-602, were found to inhibit growth of different human cancer lines and xenografted into nude mice in microgram doses after subcutaneous administration^15,17,18^. The most potent antitumor analogs, MIA-690 and MIA-602 also showed antinflammatory activities^15^. However the MIA-series of GHRH analogs with increased GHRH-R binding affinity have a weak GH inhibitory activity on pituitary somatotrophs^15^. GHRH agonists of MR series, such as MR-409, exhibit higher potency upon subcutaneous administration and binding activity than the parent hormone^14,17^. Recently, MR-409, a GHRH agonist, was found to inhibit *in vivo* growth of lung cancer xenografted into nude mice^14,16^. The antinflammatory and antioxidant properties of MR-409 could be implicated in these effects. In addition, a GHRH agonist, JI-34, was found to induce anxiety and depression whereas MZ-4-71, a GHRH antagonist, elicited anxiolytic and anti-depressant effects^19,20^. In addition, we previously found that mice with GH deficiency due to removal of GHRH gene (GHRHKO) had decreased anxiety- and depression-related behaviour^21^. The aim of our work was to investigate the potential anti-inflammatory and antioxidant effects of GHRH antagonist MIA-690 and agonist MR-409 in the brain, and the role of both classes of analogs on emotional behavior in adult mice.

## Results

### Inhibitory effects of MIA-690 and MR-409 on LPS-induced prostaglandin (PG)E_2_ and 8-iso-PGF_2α_ levels in prefrontal cortex specimens

**Figure 1:**
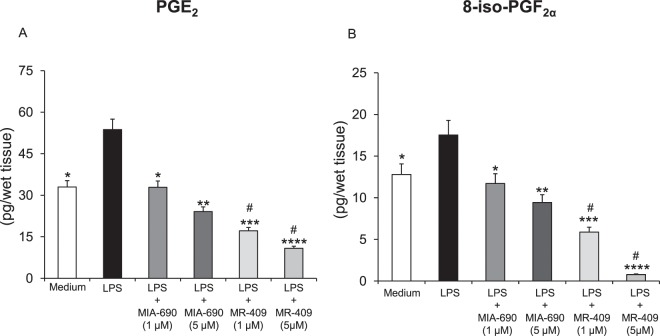
Inhibitory effects of MIA-690 (1–5 μM) and MR-409 (1–5 μM) on PGE_2_ and 8-iso-PGF_2α_ levels, *ex vivo* (n = 5 for each group of treatment). Data are expressed as means ± S.E.M. and analyzed by analysis of variance (ANOVA) followed by Bonferroni *post-hoc* test *p < 0.05, **p < 0.01,***p < 0.005; ****p < 0.001 vs. LPS group; ^#^p < 0.05 vs. co-respective treatment with MIA-690.

Tissue supernatants PGE_2_ and 8-iso-PGF_2α_ levels were determined by radioimmunoassay (RIA), after treatment of prefrontal cortex specimens with LPS + MIA-690 (1–5 μM), LPS + MR-409 (1–5 μM), LPS or vehicle. Treatment with LPS induced a significant increase of PGE_2_ and 8-iso-PGF_2α_ levels in prefrontal cortex specimens, as compared to vehicle treated controls. The GHRH antagonist MIA-690 (1–5 μM) and GHRH agonist MR-409 (1–5 μM) were found to inhibit LPS-induced PGE_2_ and 8-iso-PGF_2α_ levels in a dose-dependent manner [Fig. 1 panel A and B; F_2/12_ = 3.11, p < 0.05 and F_2/12_ = 6.93, p < 0.01 (for MIA-690); F_2/12_ = 5.10, p < 0.005 and F_2/12_ = 12.97, p < 0.001 (for MR-409)]. In this context, MR-409 (1–5 μM) was more effective than MIA-690 in decreasing LPS-induced PGE_2_ and 8-iso-PGF_2α_ levels [Fig. 1 panel A and B; F_2/12_ = 3.11, p < 0.05].

### Inhibitory effects of MIA-690 and MR-409 on LPS-induced lactate dehydrogenase (LDH) and nitrite production in prefrontal cortex specimens

**Figure 2:**
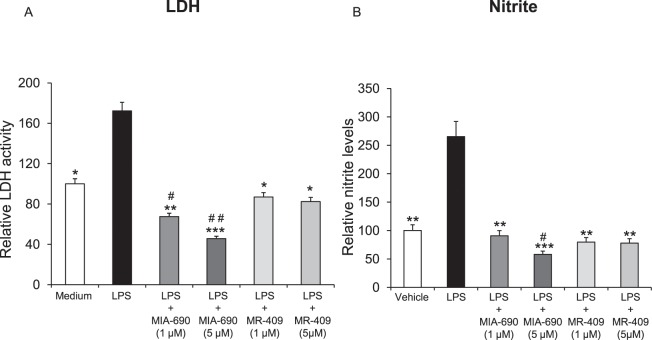
Inhibitory effects of MIA-690 (1–5 μM) and MR-409 (1–5 μM) on LDH and nitrite production, *ex vivo* (n = 5 for each group of treatment). Data are expressed as means ± S.E.M. and analyzed by analysis of variance (ANOVA) followed by Bonferroni *post-hoc* test *p < 0.05, **p < 0.01,***p < 0.005 vs LPS group; ^#^p < 0.05 and ^##^p < 0.005 vs. co-respective treatment with MR-409.

In order to evaluate potential effects of MIA-690 (1–5 μM) and MR-409 (1–5 μM) on oxidative stress biomarkers, we measured LPS-induced LDH and nitrite production in prefrontal cortex specimens treated with the peptides. LPS treatment induced a significant increase of LDH and nitrite production in prefrontal cortex specimens, as compared to vehicle treated controls. MIA-690 (1–5 μM) decreased LDH activity and nitrite levels in a dose-dependent manner [Fig. 2 panel A and B; F_4/14_ = 5.04, p < 0.01 and F_4/14_ = 3.89, p < 0.005]. Similarly, MR-409 (1–5 μM) inhibited LPS-induced LDH activity and nitrite levels, without showing a dose-dependent effect [Fig. 2 panel A and B; F_4/12_ = 4.47, p < 0.05 and F_4/12_ = 5.41, p < 0.01]. MIA-690 (1–5 μM) was more effective in decreasing LPS-induced LDH and nitrite production compared to MR-409 [Fig. 2 panel A and B; F_4/14_ = 3.11, p < 0.05 and F_4/14_ = 3.89, p < 0.005].

### MIA-690 and MR-409 decrease LPS- induced cyclooxygenase-2 (COX-2), nuclear factor-kB (NF-kB) and inducible nitric oxide synthase (iNOS) gene expression in prefrontal cortex specimens

**Figure 3:**
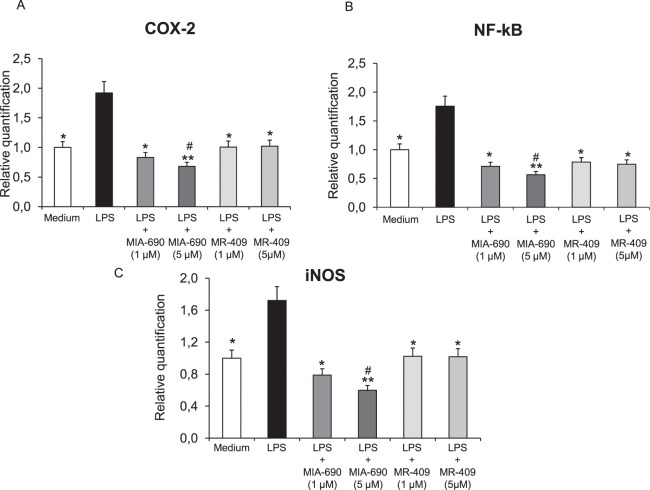
Relative quantification of COX-2, NF-kB and iNOS gene expression in mouse prefrontal cortex specimens treated with MIA-690 (1–5 μM) and MR-409 (1–5 μM), *ex vivo* (n = 5 for each group of treatment). Data were calculated using the 2^−ΔΔCt^ method, normalized to β-actin mRNA levels, and expressed relative to control (calibrator sample, defined as 1.00). Data are expressed as means ± S.E.M. and analyzed by analysis of variance (ANOVA) followed by Bonferroni *post-hoc* test *p < 0.05, **p < 0.01 vs. LPS group; ^#^p < 0.05 vs. co-respective treatment with MR-409.

Real-time polymerase chain-reaction (PCR) revealed a significantly increase in COX-2, NF-kB and iNOS gene expression in prefrontal cortex specimens after LPS treatment, as compared to vehicle treated controls. The GHRH antagonist MIA-690 (1–5 μM) inhibited LPS-induced inflammatory markers in a dose-dependent manner in prefrontal cortex specimens [Fig. 3 panel A, B and C; F_6/18_ = 2.66, p < 0.05 and F_6/18_ = 6.01, p < 0.01]. Our findings also showed that the GHRH agonist MR-409 (1–5 μM) inhibited LPS-induced COX-2, NF-kB and iNOS gene expression in prefrontal cortex specimens, without a dose-dependent effect [Fig. 3 panel A, B and C; F_5/15_ = 2.90, p < 0.05]. MIA-690 (5 μM) was more effective than MR-409 in decreasing all the markers tested [Fig. 3 panel A, B and C; F_6/18_ = 2.66, p < 0.05].

### Exploration behavioral analysis

**Figure 4:**
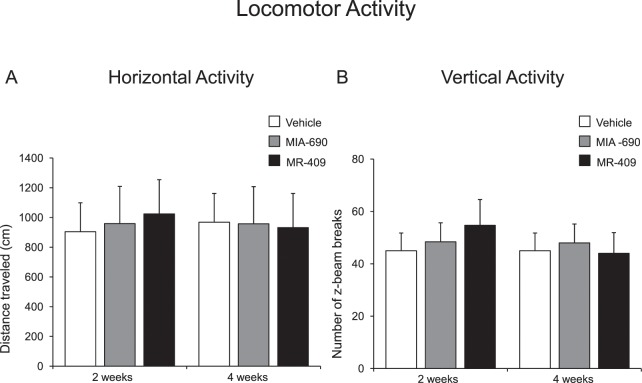
Locomotor activity in mice treated with MIA 690 (5 μg) and MR 409 (5 μg) (n = 18 for each group of treatment). Compared to vehicle, MIA-690 and MR-409 treatment did not change locomotor activity. Horizontal activity (**A**) and vertical activity (**B**) were recorded for 10 min. Data are expressed as means ± S.E.M. and analyzed by analysis of variance (ANOVA) followed by Bonferroni *post-hoc* test.

Horizontal and vertical activity was recorded in the home cage over 10 min. MIA-690 (5 μg) or MR-409 (5 μg) was s.c. injected daily for 4 weeks in mice. Control animals received s.c. injection of vehicle [0.1% DMSO (Sigma) and 10% propylene glycol]. As shown in Fig. 4, s.c. administration of MIA-690 (5 μg) and MR-409 (5 μg) did not modify locomotor activity respect to vehicle injected animals. Two-way ANOVA did not show significant differences in horizontal (Fig. 4 panel A; 2 wk F_2/27_ = 3.27, p = 0.57; 4 wk F_2/27_ = 0.11, p = 0.89) and vertical activity (Fig. 4 panel B; 2 wk F_2/27_ = 3.15, p = 0.058; 4 wk F_2/27_ = 0.57, p = 0.56) at 2 and 4 weeks of treatment, with respect to controls.

#### Anxiety-like behavior

**Figure 5:**
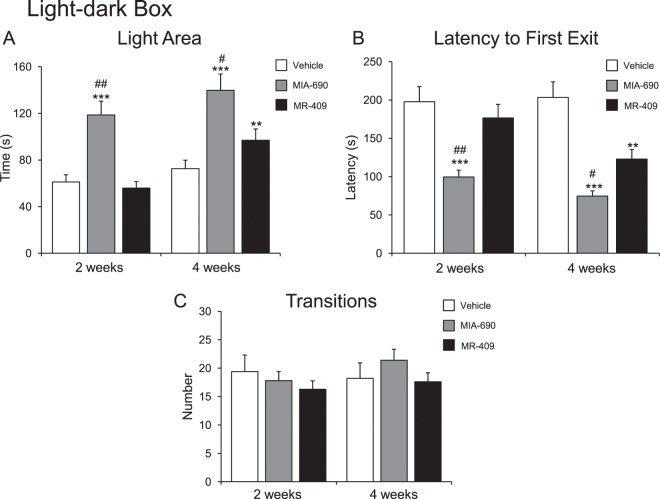
Analysis of anxiety-related behavior in mice treated with MIA-690 (5 μg) or MR-409 (5 μg) (n = 18 for each group of treatment). Compared to vehicle, MIA-690 (2–4 wk) and MR-409 (4 wk) decreased anxiety-like behavior in light-dark box. MIA-690 was more effective than MR-409 (2–4 wk). Data are expressed as means ± S.E.M. and analyzed by analysis of variance (ANOVA) followed by Bonferroni *post-hoc* test **p < 0.01,***p < 0.005 vs. control; ^#^p < 0.05 and ^##^p < 0.005 vs. MR-409 treated mice.

**Figure 6:**
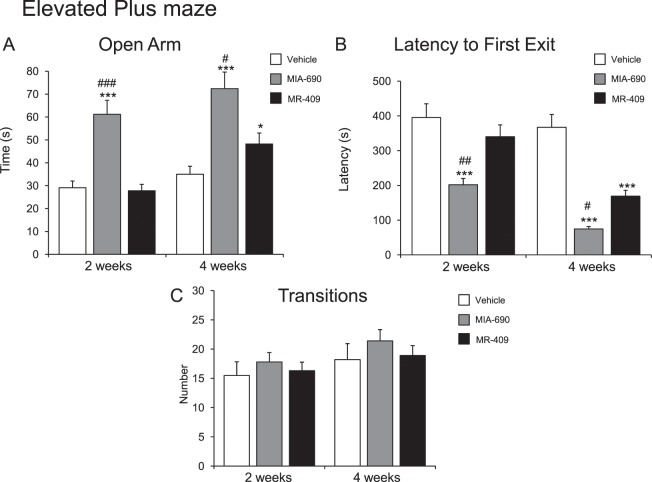
Analysis of anxiety-related behavior in mice treated with 5 μg MIA-690 or 5 μg MR-409 (n = 18 for each group of treatment). Compared to the vehicle, MIA-690 (2–4 wk) and MR-409 (4 wk) decreased anxiety-like behavior in elevated plus maze test. MIA-690 was more effective than MR-409 (2–4 wk). Data are expressed as means ± S.E.M. and analyzed by analysis of variance (ANOVA) followed by Bonferroni *post-hoc* test *p < 0.05, ***p < 0.005 vs. control; ^#^p < 0.05, ^##^p < 0.005 and ^###^p < 0.001 vs. MR-409 treated mice.

To evaluate the possible effects of MIA-690 (5 μg) and MR-409 (5 μg) on anxiety-like behavior, light-dark box and elevated plus maze test were used. The evaluations were performed at 2 and 4 weeks of treatment. We found that s.c. injection of MIA-690 (5 μg) or MR-409 (5 μg) decreased anxiety related behavior (Figs. 5 and 6) at 2 and 4 weeks of treatment, for MIA-690, and at 4 weeks of treatment for MR-409. Compared to vehicle, treatment with MIA-690 and MR-409 increased time spent in the light area [Fig. 5 panel A; 2 wk F_9/9_ = 2.73, p < 0.005; 4 wk F_9/9_ = 3.33, p < 0.005 (for MIA-690); 4 wk F_9/9_ = 1.64 p < 0.01 (for MR-409)] and open arms [Fig. 6 panel A; 2 wk F_9/9_ = 3.30, p < 0.005; 4 wk F_9/9_ = 2.56, p < 0.005 (for MIA-690); 4 wk F_9/9_ = 1.47, p < 0.05 (for MR-409)] in light-dark and elevated plus maze, respectively. Both peptides decreased latencies to emerge from enclosed dark compartment in the light-dark box [Fig. 5 panel B; 2 wk F_9/9_ = 2.21, p < 0.005; 4 wk F_9/9_ = 1.76 p < 0.005(for MIA-690); 4 wk F_9/9_ = 1.11, p < 0.01 (for MR-409)] and from the central zone in the elevated plus maze [Fig. 6 panel B; 2 wk F_9/9_ = 3.46, p < 0.005; 4 wk F_9/9_ = 23.61, p < 0.005 (for MIA-690); 4 wk F_9/9_ = 6.35, p < 0.01 (for MR-409)]. General activity, measured as the number of the total transitions, was not changed in both tests [Fig. 5 panel C; 2 wk F_9/9_ = 2.53, p = 0.18; 4 wk F_9/9_ = 3.61, p = 0.6 (for MIA-690); 2 wk F_9/9_ = 2.33, p = 0.22; 4 wk F_9/9_ = 1.45, p = 0.58 (for MR-409); Fig. 6 panel C; 2 wk F_9/9_ = 1.45, p = 0.58; 4 wk F_9/9_ = 3.61, p = 0.6 (for MIA-690); 2 wk F_9/9_ = 1.34, p = 0.6; 4 wk F_9/9_ = 1.58, p = 0.51 (for MR-409)].

In both tests, MIA-690 was more effective than MR-409 in decreasing anxiety related behavior [Fig. 5; 2 wk F_2/27_ = 26.87, p < 0.005; 4 wk F_2/27_ = 23.31, p < 0.05 (light area) and 2 wk F_2/27_ = 76.75, p < 0.005; 4 wk F_2/27_ = 23.31, p < 0.05 (latency to first exit) for light-dark exploration test] [Fig. 6; 2 wk F_2/27_ = 28.88, p = 0.001; 4 wk F_2/27_ = 30.59, p = 0.05 (open arms) and 2 wk F_2/27_ = 40.17, p < 0.005; 4 wk F_2/27_ = 76.64, p < 0.05 (latency to first exit) for elevated plus maze test].

#### Behavioral despair

**Figure 7:**
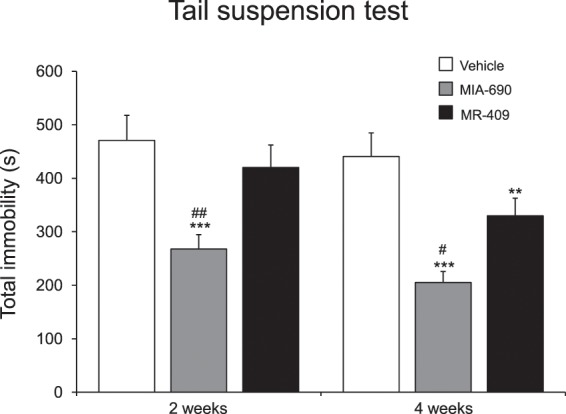
Behavioral despair measured in tail suspension test in mice treated with 5 μg MIA-690 and 5 μg MR-409 (n = 18 for each group of treatment). Compared to vehicle, MIA-690 (2–4 wk) and MR-409 (4 wk) decreased behavioral despair. MIA-690 was more effective than MR-409 (2–4 wk). Data are expressed as means ± S.E.M. and analyzed by analysis of variance (ANOVA) followed by Bonferroni *post-hoc* test **p < 0.01, ***p < 0.005 vs. control; ^#^p < 0.05 and ^##^p < 0.005 vs. MR-409 treated mice.

To evaluate the possible effects of MIA-690 (5 μg) and MR-409 (5 μg) on behavioral despair, the tail suspension test was used. The evaluations were performed at 2 and 4 weeks of treatment. Figure 7 shows total immobility time in tail suspension test. MIA-690 (5 μg) and MR-409 (5 μg) s.c. injection induced a significant decrease of total immobility (Fig. 7) at 2 and 4 weeks of treatment, for MIA-690, and at 4 weeks of treatment for MR-409 [Fig. 7; 2 wk F_9/7_ = 11.10, p < 0.005; 4 wk F_9/7_ = 2.01, p < 0.005 (for MIA-690); 4 wk F_9/7_ = 16.14, p = 0.01 (for MR-409)]. In the tail suspension test, MIA-690 was more effective than MR-409 on inducing immobility (Fig. 7; 2 wk F_2/23_ = 52.48, p < 0.005; 4 wk F_0/0_ = 51.59, p = 0.05).

#### Monoamine levels in prefrontal cortex

To further evaluate the possible mechanisms involved in emotional behavior, we measured monoamine levels in prefrontal cortex by HPLC. Table 1 shows an increase in norepinephrine (NE) and serotonin (5-hydroxytryptamine, 5-HT) levels in prefrontal cortex of mice treated with MIA-690 (5 μg) and MR-409 (5 μg), [F_3/9_ = 6.99, p < 0.005 and F_3/9_ = 13.90, p < 0.001] without any affect on dopamine (DA) levels, as compared to controls [F_2/27_ = 3.27, p = 0.57]. In addition, the increase in NE and 5-HT levels was greater with MIA-690 respect to MR-409 [F_3/9_ = 3.86, p < 0.005].

**Table 1:** Aminergic neurotransmitter levels (ng/mg wet tissue) in prefrontal cortex.

	NE	DA	5-HT
Vehicle	0.09 ± 0.02	0.70 ± 0.18	0.34 ± 0.02
MIA-690	0.85 ± 0.05^***,^^##^	0.72 ± 0.00	6.38 ± 1.32^***,^^##^
MR-409	0.32 ± 0.50^**^	0.77 ± 0.11	4.19 ± 0.23^**^

Data are expressed as means ± S.E.M. and subjected to analysis of variance (ANOVA) followed by Bonferroni *post-hoc* test **p < 0.005,***p < 0.001 vs. vehicle; ^##^p < 0.005 vs. MR-409 treated mice.

### MIA-690 and MR-409 decreased NF-kB, tumor necrosis factor-α (TNF-α) and interleukin (IL)-6 gene expression in mice prefrontal cortex

**Figure 8:**
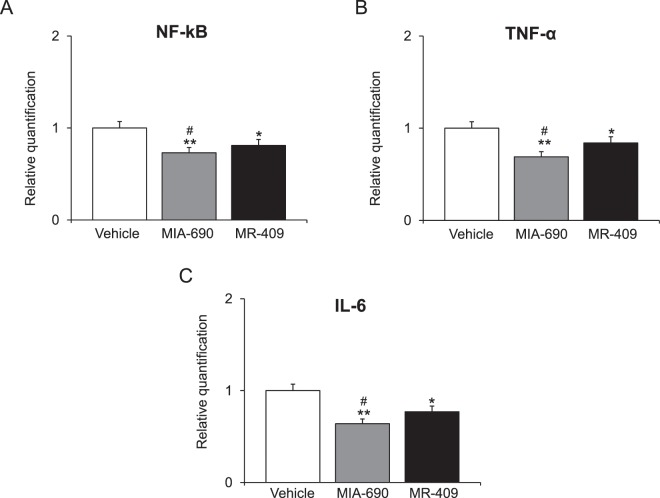
Relative quantification of gene expression of NF-kB, TNF-α and IL-6 (n = 9 for each group of treatment) in prefrontal cortex. Data were calculated using the 2^−ΔΔCt^ method, normalized to β-actin mRNA levels, and expressed relative to control (calibrator sample, defined as 1.00). Data are expressed as means ± S.E.M. and analyzed by analysis of variance (ANOVA) followed by Bonferroni *post-hoc* test *p < 0.05 **p < 0.01 vs. vehicle group; ^#^p < 0.05 vs. MR-409 treated mice.

Real-time polymerase chain-reaction (PCR) revealed a significant decrease in NF-kB, TNF-α and IL-6 gene expression after MIA-690 (5 μg) and MR-409 (5 μg) treatment in prefrontal cortex, in mice [F_2/16_ = 2.85, p < 0.05 and F_2/16_ = 10.97, p < 0.001]. MIA-690 was more effective than MR-409 in decreasing NF-kB, TNF-α and IL-6 [Fig. 8 panel A, B and C; F_5/16_ = 2.85, p < 0.05].

### Haematoxylin-eosin staining and immunohistochemical analysis of nuclear factor erythroid 2–related factor 2 (Nrf2) in mouse prefrontal cortex

**Figure 9:**
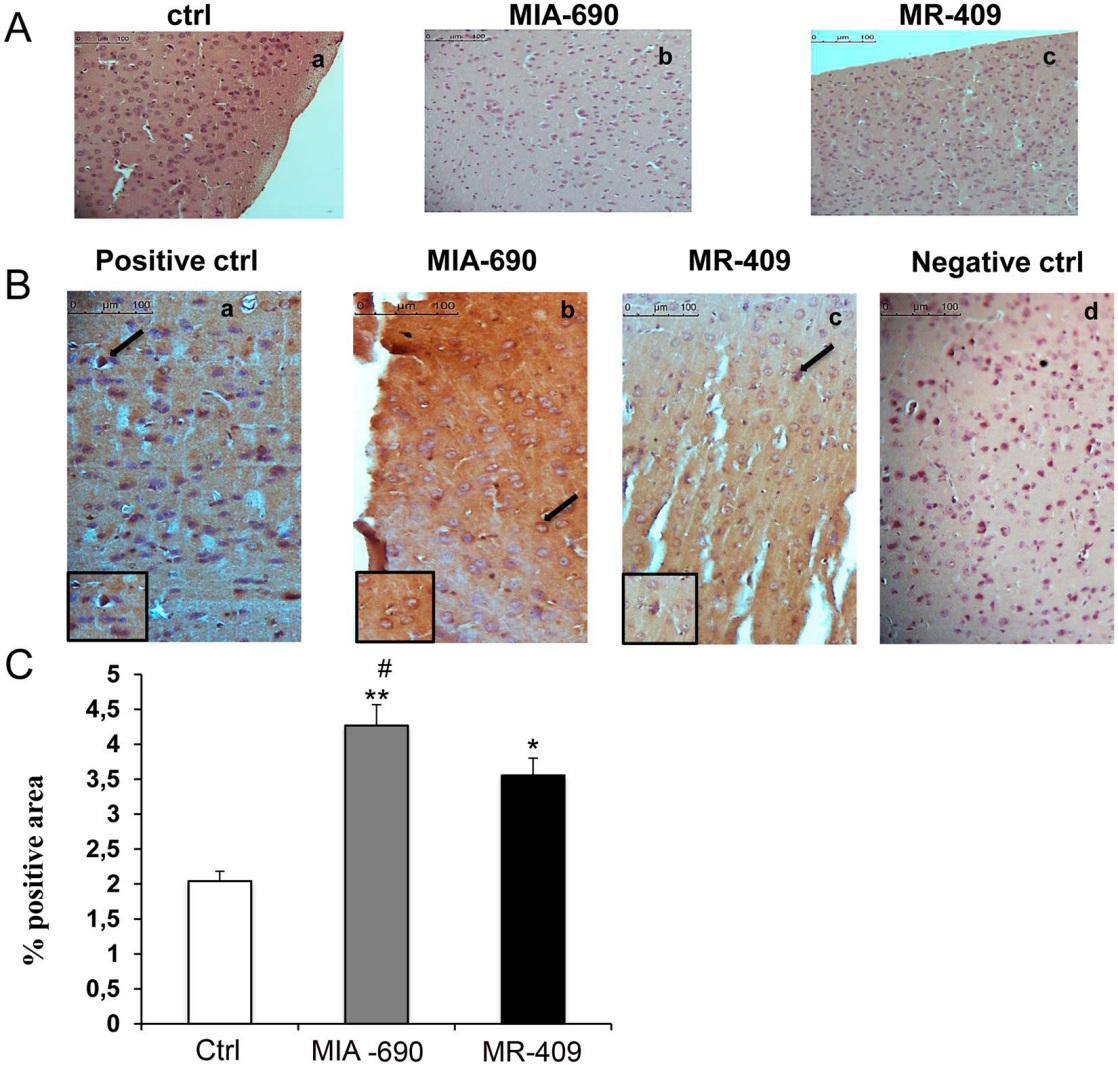
Haematoxylin-eosin staining and immunohistochemical analysis of Nrf2 expression in mouse prefrontal cortex exposed to subcutaneous chronic treatment for 4 weeks (n = 9 for each group of treatment). (**A**) Haematoxylin-eosin staining (a) positive control (ctrl); (b) mice treated with MIA-690; (c) mice treated with MR-409. Scale bar: 100 µm, magnification 20x. (**B**) Immunohistochemical detection of Nrf2 expression in mice exposed to subcutaneous chronic treatment (a) positive control (ctrl); (b) mice treated with MIA-690; (c) mice treated with MR 409; (d) negative ctrl; Insert shows Nrf2 nuclear staining; arrows indicate Nfr2 positive area. Scale bar: 100 µm, magnification 20x. (**C**) Graphic representation of the percentage of Nrf2 positive area ( ± SD); densitometric analysis determined by direct visual counting of ten fields for each of three slides per sample. *p < 0.05 and **p < 0.01 vs. ctrl; ^#^p < 0.01 vs. MR-409.

Morphological features and detection of Nrf2 in prefrontal cortex have been analyzed by hematoxylin-eosin (H&E) staining and immunohistochemistry, respectively. H&E stained sections of the (a) control (ctrl), (b) MIA-690 and (c) MR-409 mice showed the normal histological structure of prefrontal cortex. The frontal cortex appeared laminated with six different layers of variable thickness that are blended with each other (Fig. 9, panel A: a, b and c).

Immunohistochemical examination revealed positive immunostaining for Nrf2 expression in mice exposed for 4 weeks to subcutaneous chronic treatment (a) positive vehicle (ctrl); (b) mice treated with MIA-690; (c) mice treated with MR-409 (Fig. 9, panel B: a, b, c and d). As compared to the control, increased Nrf2 immunostaining was detected in mice treated with MIA-690 or MR-409 [Fig. 9 panel C; F_2/15_ = 2.79, p < 0.05 and F_2/15_ = 11.34, p < 0.01]. Our findings also showed that MIA-690 increased immunoreactivity for Nrf2 respect to MR-409 [Fig. 9 panel C; F_2/15_ = 6.36, p < 0.01].

### MR-409 induced down regulation of P GHRH-R gene and protein expression in prefrontal cortex

**Figure 10:**
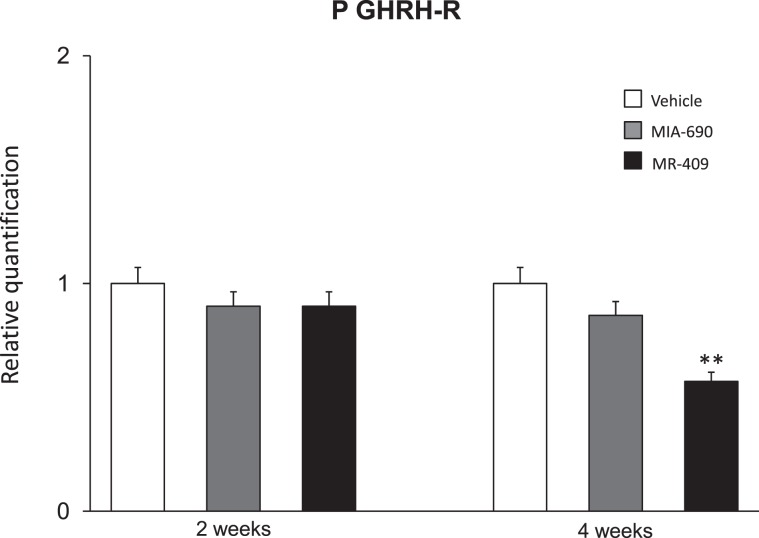
Relative quantification of P GHRH-R gene expression (n = 9 for each group of treatment). Data were calculated using the 2^−ΔΔCt^ method, normalized to β-actin mRNA levels, and expressed as relative to control (calibrator sample, defined as 1.00). Data are expressed as means ± S.E.M. and analyzed by analysis of variance (ANOVA) followed by Bonferroni *post-hoc* test **p < 0.005 vs. vehicle and MIA-690 treated mice.

**Figure 11:**
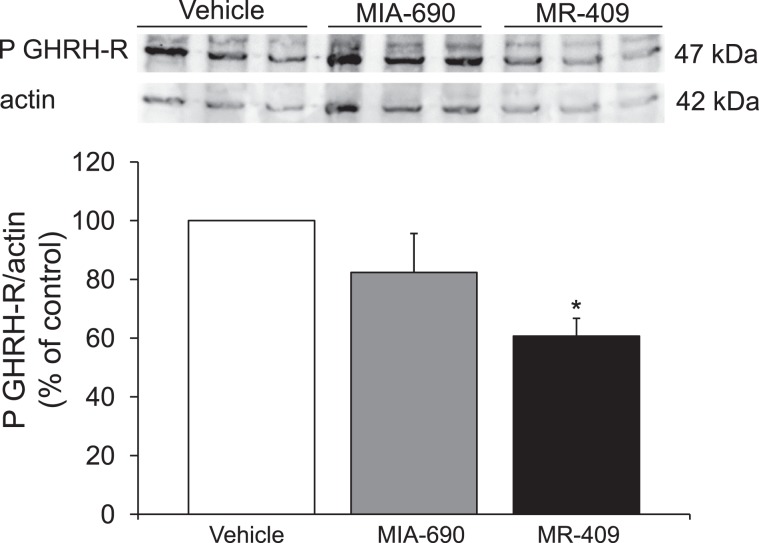
Protein expression for P GHRH-R in mouse prefrontal cortex exposed to subcutaneous chronic treatment for 4 weeks (n = 3 for each group of treatment), assessed by Western blot. Actin served as internal control. Data are expressed as means ± S.E.M. and analyzed by analysis of variance (ANOVA) followed by Bonferroni *post-hoc* test.*p < 0.05 vs vehicle. The grouping of gels is cropped from different parts from different gels.

We finally evaluated the effects of MIA-690 (5 μg) and MR-409 (5 μg) treatment on P GHRH-R gene and protein expression in prefrontal cortex. Compared to vehicle treated mice, subcutaneous injection of MR-409 induced a significant reduction in P GHRH-R gene and protein expression in prefrontal cortex after 4 weeks of treatment [Fig. 10; F_2/15_ = 2.79, p < 0.005; Fig. 11; F_2/6_ = 0.79, p < 0.05] (Supplementary Figs. S1 and S2).

## Discussion

GHRH and mRNAs for GHRH-R have been found in rat cortex and brain stem^22,23^, and various reports suggested that GHRH might play a key role in cognitive and mood disorders^20,24–28^. GHRH-antagonists compounds can exert powerful antitumor effects, possibly related in part to their antinflammatory and antioxidant properties^17,29–33^. In the present study we show that MIA-690, a GHRH antagonist, and MR-409, a GHRH analog, exhibit antinflammatory and antioxidant effects on prefrontal cortex specimens, *ex vivo* (Figs. 1, 2 and 3). Accordingly, various studies showed that GHRH and GHRH antagonists can influence the inflammatory and reduction/oxidation (redox) status in cancer and other tissues^29,33^.

In particular, MIA 690 decreased inflammation by reducing the infiltration of macrophages and leucocytes, the production of TNF-α, IL-1β, and monocyte chemotactic protein-1 (MCP-1) in tissue after insult with lipopolisaccaride (LPS) and the production of the pro-inflammatory markers in carrageenan-induced chronic prostatis^31,32^. In addition, MIA-690 showed antioxidant and neuroprotective properties^28^. In a model of Alzheimer’s disease as well as in cancer and other tissues, MR-409 has been described to exert antinflammatory and antioxidant effects, as well as in early experimental diabetic retinopathy^31^. In this context, the authors suggested that the protective effects of the MR-409 could be mediated by its direct and/or GH-mediated action.

In the present study, MIA-690 and MR-409 have been also able to modulate emotional behaviors, in mice. We observed that both peptides induced anxiolytic and antidepressant-like effects following chronic treatment, without affecting locomotor activity (Figs. 4–7). The role of GH in regulation of mood is somewhat contradictory and little is known about the action of GHRH on brain functions. Human studies indicate that in adults with childhood onset GH deficiency, long term treatment with GH improves mood and memory^34^. On the other hand, somatostatin, which inhibits the release of several hormones, including GH, reduces anxiety-like behavior^35,36^. Mood disorders might be related to GH deficiency^37,38^, however the anxiolytic-antidepressant effects of a GHRH antagonist, MZ-4-71, suggests that GHRH itself may be involved in control of behavior^20,25–27^. Similarly, our research group described anxiolytic and antidepressant-like behavior in both young and old mice with generalized ablation of the GHRH gene^21,39,40^. Thus, we can hypothesize that the beneficial behavioral effects of MIA-690 and MR-409 could be at least in part related to their antinflammatory and antioxidant effects (Figs. 8, 9), also described in different reports^14,30–33^. On the other hand, inflammation and oxidative stress are linked to a number of chronic diseases, including cancer, cardiovascular diseases, aging, neurodegenerative, and psychiatric disorders, such as anxiety and depression^40–43^. The activation of the inflammatory and oxidative stress response leads to the release of inflammatory cytokines and mobilization of immune cells that can get access to brain^42,43^. In particular, some studies have demonstrated an increase in pro-inflammatory markers, such as NF-kB, IL-1 and IL-6, in anxiety- and depression-related conditions^44–46^. In addition, major depression and posttraumatic stress disorder are characterized by an increased activity of pro-oxidants over antioxidants^47,48^. Cytokines and their signaling pathways have significant effect on the metabolism of multiple neurotransmitters such as 5-HT and DA through impact on their synthesis, release and reuptake. Through their effects on neurotransmitter systems, cytokines lead to significant changes in motor activity and motivation as well as anxiety, arousal and alarm^49^. In this context, we also evaluated monoamine levels and NF-kB, TNF-α and IL-6 gene expression in prefrontal cortex of mice, after chronic administration with MIA-690 and MR-409. We observed an increase of NE and 5-HT levels, paralleled by a decrease of inflammatory markers in both MIA-690 and MR-409 treated mice (Table 1; Fig. 8, panel A, B and C). In agreement, the anxiolytic-antidepressant activity of MZ-4-71 was previously suggested to be mediated by α1/α2-adrenergic and 5-HT1/5-HT2 serotonergic receptors^25,26^. Because several studies established a link between oxidative stress, anxiety and depression, we investigated the expression of Nrf2 in prefrontal cortex by immunohistochemistry. Nrf2 is a key transcription factor controlling various homoeostatic processes, at cellular level, in response to oxidative stress and toxic stimuli^50^ and regulating oxidative/xenobiotic stress response, also repressing inflammation^51^. Activation of Nrf2 results in up-regulation of cytoprotective and antioxidant enzymes-proteins in brain, by protecting against oxidative stress, in the brain^50,51^. Less Nfr2 signaling could reflect a “normalization” of oxidative parameters also evidenced from the molecular markers.

We found a significant increase of positive area percentage for Nrf2 in MIA-690 or MR-409 treated mice, suggesting an evident Nrf2 activation (Fig. 9). Surprisingly, both the agonist and the antagonist peptides induced similar effects in all experimental paradigms. However, we observed a higher efficacy of MIA-690 compared to MR-409. An accurate behavior analysis showed that MIA-690 was able to modulate emotional behavior beginning from week 2 of treatment, while MR-409 induced significant behavioral change only at week 4 of treatment. At the end of treatment, we found a reduction of P GHRH-R gene and protein expression in MR-409 treated mice (Figs. 10, 11) (Supplementary Figs. S1 and S2). As previously observed by Schally *et al*.^16^, our results confirmed that chronic administration of MR-409 results in a down-regulation of P GHRH-R, which could justify the effects of MR-409 on emotional behavior. On the other hand, we can not rule out the possible involvement of other mechanisms in emotional behavior induced by the peptide.

In conclusion, both MIA-690 and MR-409 exhibit antinflammatory and antioxidant effects in *ex vivo* and *in vivo* experimental models. Surprisingly, both agonist and antagonist peptides induce anxiolytic and antidepressant-like behavior, which could be related to increased cortical NE and 5-HT levels, along with modulatory effects on the inflammatory and oxidative status. Further investigations are needed to confirm a role for GHRH analogs in mood disorders.

## Methods

### Peptides and chemicals

The GHRH antagonist MIA-690 and agonist MR-409 were synthesized by R.C. and W.S. in the laboratory of one of us (A.V.S.). For *ex vivo* studies, the peptides were dissolved in DMSO to form a 5 mM solution, and then further diluted to the concentration indicated. For *in vivo* studies, the peptides were dissolved in an aqueous solution of 0.1% DMSO (Sigma) and 10% propylene glycol (Sigma-Aldrich, St. Louis, MO)^16,28^.

### Animals

Adult C57/BL6 male mice (3 month- old, weight 20–25 g, n = 48) were housed in plexiglas cages (2–4 animals per cage; 55 × 33 × 19 cm) and maintained under standard laboratory (21 ± 2 °C; 55 ± 5% humidity) on a 14/10 h light/dark cycle, with ad libitum access to water and food. Only male mice were used to avoid any possible involvement of hormonal changes in adult female mice. Mice were fed with a standard rodent chow (Prolab RMH2500, PMI Nutrition International, Brentwood, MO). Housing conditions and experimentation procedures were strictly in agreement with the European Community ethical regulations (EU Directive n. 26/2014) on the care of animals for scientific research. In agreement with the recognized principles of “Replacement, Refinement and Reduction of Animals in Research”, prefrontal cortex specimens were obtained as residual material from vehicle-treated mice randomized in our previous experiments approved by Local Ethical Committee (‘G. d’Annunzio’ University, Chieti-Pescara) and Italian Health Ministry (Project n. 885/2018-PR).

### *Ex vivo* studies

Mice were sacrificed by CO_2_ inhalation (100%CO_2_ at a flow rate of 20% of the chamber volume per min), then brains were rapidly removed. The brains were cut into blocks containing the entire prefrontal cortex, frozen on dry ice, and stored at −80 °C before serial cryosectioning at a section thickness of 100 *μ*m. A stereotaxic atlas of the mouse brain (Paxinos and Watson) was used during the cryosectioning procedure^52^. Tissue slices were maintained in a humidified incubator with 5% CO_2_ at 37 °C for 4 h (incubation period), in RPMI buffer with added bacterial LPS (10 μg/mL), as previously described^53^. During the incubation period, the tissues were treated with MR-409 or MIA-690 (1–5 μM). Tissue supernatants were collected and PGE_2_ and 8-iso-PGF_2α_ levels (pg/mg wet tissue) were measured by RIA, as previously reported^54^. Briefly, specific anti-PGE_2_ and anti-8-iso-PGF_2α_ were developed in the rabbit; the cross-reactivity against other prostanoids was <0.3%. One hundred microliters of prostaglandin standard or sample was incubated overnight at 4 °C with the ^3^H-prostaglandin (3000 cpm/tube; NEN) and antibody (final dilution: 1:120 000; kindly provided by the late prof. G. Ciabattoni), in a volume of 1.5 mL of 0.025 M phosphate buffer. Free and antibody-bound prostaglandins were separated by the addition of 100 μL 5% bovine serum albumin and 100 μL 3% charcoal suspension, followed by centrifugation for 10 min at 4000 *g* at 5 °C and decanting off the supernatants into scintillation fluid (UltimaGold™, Perkin Elmer) for β emission counting. The detection limit of the assay method was 0.6 pg/mL.

Tissue supernatants were also assayed for nitrite determination by Griess assay, as previously described^55^. Briefly, nitrite production was determined by mixing 50 μL of the assay buffer with 50 μL of Griess reagent (1.5% sulfanilamide in 1 M HCl plus 0.15% N-(1-naphthyl) ethylenediamine dihydrochloride in distilled water, v/v). After incubation for 10 min, at room temperature, the absorbance at 540 nm was determined and nitrite concentrations were calculated from a standard curve for sodium nitrite.

Tissue supernatants were also assayed for lactate dehydrogenase (LDH) activity^55^. LDH activity was measured by evaluating the consumption of NADH in 20 mM HEPES-K + (pH 7.2), 0.05% bovine serum albumin, 20 μM NADH and 2 mM pyruvate using a microplate reader (excitation 340 nm, emission 460 nm) according to manufacturer’s protocol (Sigma-Aldrich, St. Louis, MO). LDH activity was measured by evaluating the consumption of NADH in 20 mM HEPES-K + (pH 7.2), 0.05% bovine serum albumin, 20 μM NADH and 2 mM pyruvate using a microplate reader (excitation 340 nm, emission 460 nm) according to manufacturer’s protocol. Nitrite and LDH production data were expressed as relative variations compared to vehicle-treated specimens. Immediately after sacrifice, prefrontal cortex was rapidly removed, dissected and stored in RNAlater solution (Ambion, Austin, TX) at −20 °C until further processed. Total RNA was extracted from the prefrontal cortex using TRI Reagent (Sigma-Aldrich), according to the manufacturer’s protocol. One microgram of total RNA extracted from each sample in a 20 μl reaction volume was reverse transcribed using High Capacity cDNA Reverse Transcription Kit (Applied Biosystems). The samples were incubated in a 2720 Thermal Cycler (Applied Biosystems) initially at 25 °C for 10 min, then at 37 °C for 120 min, and finally at 85 °C for 5 s. Gene expression of COX-2, NF-kB and iNOS was determined by quantitative real-time PCR using TaqMan probe-based chemistry (Applied Biosystems), as previously described^56,57^. PCR primers and TaqMan probes were obtained from Applied Biosystems (Assays-on-Demand Gene Expression Products, Mm00478374_m1 for COX-2 gene, Mm00476361_m1 for NF-kB gene, Mm00440502_m1 for iNOS gene, Mm00607939_s1 for β-actin gene. β-actin was used as the housekeeping gene. Gene expression data were calculated as previously reported^58^.

### *In vivo* studies

After 2-week acclimation, mice were randomized into three groups and treated daily for 4 weeks by subcutaneous administration of GHRH antagonist MIA 690 (5 μg), GHRH agonist MR 409 (5 μg) or vehicle solution^18^. All solutions were prepared freshly before use.The doses were selected based on previous studies including oncology. Injection volume was 0.1 ml for s.c. injection^16,18^. The animals were brought into the experimental room 30 min prior to the test in order to acclimate to the environment, and were kept in the testing chamber for 5 min prior to each test.

All treatments were administered at 09:00 am, and the experiments performed between 10:00 and 12:00 am. Each test session was recorded by a video camera connected to a computer; a single video frame was acquired with a highly accurate, programmable, monochrome frame grabber board (Data TranslationTM, type DT3153). The intelligent software Smart version 2.5 (Panlab, sl Bioresearch and Technology, Barcelona, Spain) was used for data processing. The apparatuses were purchased from 2 Biological Instruments (Besozzo VA, Italy)^21,39^. At the end of each test, the animals were returned to their home cages, and the apparatus was cleaned with 75% ethanol and dried before the next procedure. The behavioral parameters were recorded at 2 and then 4 weeks after the first treatment. Each test was conducted on the same group of animals (n = 18 animals for each group of treatment), after a 2 weeks rest period to avoid any interference on behavioral test performance, as previously reported^21^.

### Locomotor activity

Locomotor activity was recorded in the home cage over 10 min. The activity monitor consisted of a black and white video camera, mounted in the top-centre of a cage (35 × 20 × 13 cm), positioned in the enclosure. Measurements used to assess locomotor activity were horizontal activity and vertical activity^59^.

### Light–dark exploration test

The light–dark box test assesses bright-space related anxiety^59,60^ and consists of two compartments (10 × 15 × 20 cm, each), dark and light ones, separated by a wall pierced with an open door. The dark compartment has opaque black walls, while the light compartment is transparent to light. Mice were placed in the black compartment, and time spent by the animal in the light compartment, latency of first exit from dark compartment, and number of transitions between compartments were recorded during a 10 min interval.

### Elevated plus maze test

The apparatus consisted of two open arms and two closed arms that extended from a common central platform, elevated to a height of 45 cm above floor level and mice were individually placed in the centre of the maze facing an open arm^40,61^. The time spent on open arms, the latency to first exit and the number of transitions between the arms were recorded during a 10 min test period.

### Tail suspension test

This test is well characterized for assessing antidepressant-like activity. Mice were individually suspended by the tail to a horizontal bar (at the height of 30 cm from floor) using adhesive tape. Immobility time was recorded during a 6 min period. Mice were considered immobile only when they hung passively and completely motionless^21,39^.

### Prefrontal cortex monoamine extraction and high performance liquid chromatography (HPLC) determination

Immediately after sacrifice, brains were rapidly removed and prefrontal cortex were dissected and subjected to biogenic amine extractive procedures. Thereafter, samples were analyzed by HPLC coupled to electrochemical detection consisting of ESA Coulochem III detector equipped with ESA 5014 B analytical cell (selected potentials: electrode 1:−150 mV; electrode 2: +300 mV), as previously reported^40,59^. Monoamine levels were expressed as ng/mg wet tissue.

### RNA extraction, reverse transcription and real-time reverse transcription polymerase chain reaction (PCR-RT)

Prefrontal cortex was rapidly removed, dissected and stored in RNAlater solution (Ambion, Austin, TX) at −20 °C until further processed as previously described. Gene expression of NF-kB, TNF-α, IL-6 and P GHRH-R was determined by quantitative real-time PCR using TaqMan probe-based chemistry (Applied Biosystems, Foster City, CA, USA). PCR primers and TaqMan probes were obtained from Applied Biosystems (Assays-on-Demand Gene Expression Products, Mm00476361_m1 for NF-kB gene, Mm00443258_m1 for TNF-α gene, Mm00446190_m1 for IL-6 gene, Mm01326479_m1 for P GHRH-R gene, Mm00607939_s1 for β-actin gene. β-actin was used as the housekeeping gene. Gene expression data were calculated as previously reported^58^.

### Light microscopy analysis and immunohistochemistry

Prefrontal cortex was fixed in 10% phosphate-buffered formalin for 2.5 hours. Each tissue block was dehydrated in a series of alcohol solutions of 50%, 70%, 96% and 99% and then in Bioclear. Samples were then paraffin-embedded and cut into 7 μm-thick sections. Sections were de-waxed (Bioclear and alcohol in progressively lower concentrations), rehydrated and processed for haematoxylin-eosin and for anti-Nrf2 immunohistochemical analysis according to manufacturer protocol. Primary antibody anti-Nfr2 (rabbit polyclonal, sc-722, Santa Cruz Biotechnology, CA, USA) was applied for 2 hours at room temperature and diluted 1:200 in PBS. The immunohistochemical reactions was revealed with Rabbit specific HRP/DAB detection IHC kit (ab236469). Peroxidase reaction was developed using diaminobenzidine (DAB) chromogen and nuclei were counterstained with haematoxylin. Lastly, sections were dehydrated, cleared with xylene and mounted in Bio Mount (Bio Optica, Milano, Italy). Negative control was performed by omitting the primary antibody. Samples were then observe by means of LEICA DM 4000 light microscopy (Leica Cambridge Ltd., Cambridge, UK) equipped with a Leica DFC 320 camera (Leica Cambridge Ltd.) for computerized images^62,63^.

### Western blot analysis

Cortex samples obtained from mice treated or not with MIA-690 or MR-409 were homogenized in RIPA buffer (Sigma-Aldrich), sonicated and centrifuged at 14,000 rpm (4 °C for 15 min). Total protein lysates were quantified with Bicinchoninic Acid kit (BCA) from Sigma-Aldrich. Proteins (35 μg) were separated by 10% SDS-PAGE, transferred to a nitrocellulose membrane and incubated overnight at 4 °C with the specific P GHRH-R antibody (dilution 1:500, rabbit polyclonal P GHRH-R antibody, Abcam, ab76263). Blots were reprobed with actin (dilution 1:500, mouse monoclonal actin antibody, Santa Cruz Biotechnology, sc-376421) for protein normalization. Immunoreactive proteins were visualized using horseradish peroxidase-conjugated goat anti-mouse, goat anti-rabbit or mouse anti-goat (1:4000) secondary antibodies by enhanced chemiluminescence substrate (ECL) using ChemiDoc XRS (Bio-Rad), densitometric analysis was performed with Quantity One software (Bio-Rad)^18^.

### Statistical analysis

Statistical analysis was performed using GraphPad Prism version 5.01 for Windows (GraphPad Software, San Diego, CA, USA). All data were collected from each of the animals used in the experimental procedure and means ± SEM were determined for each experimental group and analyzed by two way analysis of variance (ANOVA) followed by Bonferroni post-hoc test. F values are referring to repeated measure 2-way ANOVA. As for gene expression analysis, 1.00 (calibrator sample) was considered the theoretical mean for the comparison. Statistical significance was accepted at p < 0.05. As regards gene expression analysis, the comparative 2^−ΔΔCt^ method was used to quantify the relative abundance of mRNA and then to determine the relative changes in individual gene expression (relative quantification)^58^. Finally, as regards the animals randomized for each experimental group, the number was calculated on the basis of the ‘Resource Equation’ N = (E + T)/T (10 ≤ E ≤ 20)^64^ according to the guidelines suggested by the ‘National Centre for the Replacement, Refinement and Reduction of Animals in Research’ (NC3RS) and reported on the following web site: https://www.nc3rs.org.uk/experimental-designstatistics.

## Supplementary information

Supplementary Information 
