# Publisher Correction: Antinflammatory, antioxidant, and behavioral effects induced by administration of growth hormone-releasing hormone analogs in mice

**DOI:** 10.1038/s41598-020-61185-x

**Published:** 2020-03-11

**Authors:** Lucia Recinella, Annalisa Chiavaroli, Giustino Orlando, Claudio Ferrante, Guya Diletta Marconi, Iacopo Gesmundo, Riccarda Granata, Renzhi Cai, Wei Sha, Andrew V. Schally, Luigi Brunetti, Sheila Leone

**Affiliations:** 1 Department of Pharmacy, G. d’Annunzio University, Chieti, Italy; 2 Division of Endocrinology, Diabetes and Metabolism, Department of Medical Sciences, University of Turin and Città Della Salute e Della Scienza Hospital, Turin, 10126 Italy; 3 Veterans Affairs Medical Center, Miami, FL 33125 USA; 4 Division of Endocrinology, Diabetes and Metabolism, Department of Medicine, Miller School of Medicine, University of Miami, Miami, FL 33136 USA; 5 Division of Medical/Oncology, Department of Pathology, Sylvester Comprehensive Cancer Center, Miami, FL 33136 USA

Correction to: *Scientific Reports* 10.1038/s41598-019-57292-z, published online 20 January 2020

In the original version of this Article, the author Guya Diletta Marconi was incorrectly indexed. This error has now been corrected.

