# Supplementary material for: Antinflammatory, antioxidant, and behavioral effects induced by administration of growth hormone-releasing hormone analogs in mice
Source: Sci Rep. 2020 Jan 20;10:732. doi: 10.1038/s41598-019-57292-z (PMC6971229; doi:10.1038/s41598-019-57292-z)
Supplement: Supplementary file 1 — Supplementary Information [file 41598_2019_57292_MOESM1_ESM.docx]

**Supplementary Information for**

**Antinflammatory, antioxidant, and behavioral effects induced by administration of growth hormone-releasing hormone analogs in mice**

Lucia Recinella^a^, Annalisa Chiavaroli^a^, Giustino Orlando^a^, Claudio Ferrante^a^, Guya Diletta Marconi^a^, Iacopo Gesmundo^b^, Riccarda Granata^b^, Renzhi Cai^c,d^, Wei Sha^c,d^, Andrew V. Schally^c,d^, Luigi Brunetti^a,*^ and Sheila Leone^a,*^.

Affiliations:

^a^Department of Pharmacy, G. d'Annunzio University, Chieti, Italy.

^b^Division of Endocrinology, Diabetes and Metabolism, Department of Medical Sciences, University of Turin and Città Della Salute e Della Scienza Hospital, Turin, 10126, Italy.

^c^Veterans Affairs Medical Center, Miami, FL 33125.

^d^Division of Endocrinology, Diabetes and Metabolism, and Division of Medical/Oncology, Department of Medicine, and Department of Pathology, Miller School of Medicine, University of Miami, Miami, FL 33136 and Sylvester Comprehensive Cancer Center, Miami, FL 33136.

**Email**: luigi.brunetti@unich.it

**This PDF file includes:**

Figs. S1 and S2


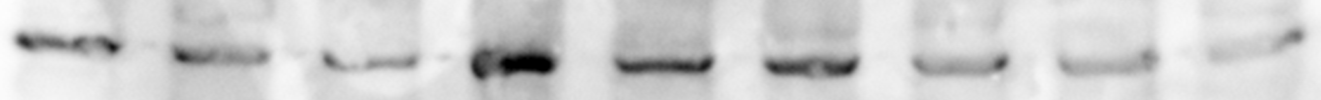


**Fig. S1**. Full-length gels for actin. Protein expression for P GHRH-R in mouse prefrontal cortex exposed to subcutaneous chronic treatment for 4 weeks (n=3 for each group of treatment), assessed by Western blot. Actin served as internal control.


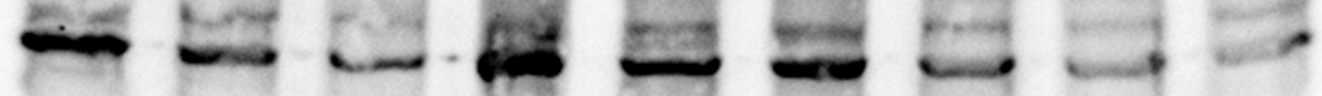


**Fig. S2**. Full-length gels for P GHRH-R. Protein expression for P GHRH-R in mouse prefrontal cortex exposed to subcutaneous chronic treatment for 4 weeks (n=3 for each group of treatment), assessed by Western blot. Actin served as internal control.
